# SnugDock: Paratope Structural Optimization during Antibody-Antigen Docking Compensates for Errors in Antibody Homology Models

**DOI:** 10.1371/journal.pcbi.1000644

**Published:** 2010-01-22

**Authors:** Aroop Sircar, Jeffrey J. Gray

**Affiliations:** 1 Chemical & Biomolecular Engineering, Johns Hopkins University, Baltimore, Maryland, United States of America; 2 Program in Molecular & Computational Biophysics, Johns Hopkins University, Baltimore, Maryland, United States of America; University of California, San Francisco, United States of America

## Abstract

High resolution structures of antibody-antigen complexes are useful for analyzing the binding interface and to make rational choices for antibody engineering. When a crystallographic structure of a complex is unavailable, the structure must be predicted using computational tools. In this work, we illustrate a novel approach, named SnugDock, to predict high-resolution antibody-antigen complex structures by simultaneously structurally optimizing the antibody-antigen rigid-body positions, the relative orientation of the antibody light and heavy chains, and the conformations of the six complementarity determining region loops. This approach is especially useful when the crystal structure of the antibody is not available, requiring allowances for inaccuracies in an antibody homology model which would otherwise frustrate rigid-backbone docking predictions. Local docking using SnugDock with the lowest-energy RosettaAntibody homology model produced more accurate predictions than standard rigid-body docking. SnugDock can be combined with ensemble docking to mimic conformer selection and induced fit resulting in increased sampling of diverse antibody conformations. The combined algorithm produced four medium (Critical Assessment of PRediction of Interactions-CAPRI rating) and seven acceptable lowest-interface-energy predictions in a test set of fifteen complexes. Structural analysis shows that diverse paratope conformations are sampled, but docked paratope backbones are not necessarily closer to the crystal structure conformations than the starting homology models. The accuracy of SnugDock predictions suggests a new genre of general docking algorithms with flexible binding interfaces targeted towards making homology models useful for further high-resolution predictions.

## Author Summary

Antibodies are proteins that are key elements of the immune system and increasingly used as drugs. Antibodies bind tightly and specifically to antigens to block their activity or to mark them for destruction. Three-dimensional structures of the antibody-antigen complexes are useful for understanding their mechanism and for designing improved antibody drugs. Experimental determination of structures is laborious and not always possible, so we have developed tools to predict structures of antibody-antigen complexes computationally. Computer-predicted models of antibodies, or homology models, typically have errors which can frustrate algorithms for prediction of protein-protein interfaces (docking), and result in incorrect predictions. Here, we have created and tested a new docking algorithm which incorporates flexibility to overcome structural errors in the antibody structural model. The algorithm allows both intramolecular and interfacial flexibility in the antibody during docking, resulting in improved accuracy approaching that when using experimentally determined antibody structures. Structural analysis of the predicted binding region of the complex will enable the protein engineer to make rational choices for better antibody drug designs.

## Introduction

High resolution structures of protein-protein complexes are necessary for understanding mechanisms of protein-protein interactions, analyzing mutations, and manipulating binding affinity [1]. The large gap between the number of experimentally determined complex structures and the available sequences of pairs of protein complexes underscores the challenges, time required and cost of x-ray crystallography or nuclear magnetic resonance approaches. The paucity in complex structures can be alleviated by computational docking, *i.e.*, the prediction of protein-protein complexes, which potentially provides a fast and efficient alternative route. To predict the structure of a protein-protein complex, computational docking requires the structures of the interacting partners. However, sometimes even the structures of the monomeric units are unavailable, forcing the use of a homology modeled structure for one or both partners [2],[3]. Given the inaccuracies in a homology model, current computational docking strategies can determine the gross structural features of a complex, but find it exceedingly challenging to successfully predict high resolution structures of such protein-protein complexes, pointing to the need to develop new docking algorithms which incorporate the necessary degrees of freedom to compensate for the inaccuracies.

Antibody-antigen (Ab-Ag) complexes provide a model system where much needed high-resolution computational docking predictions are challenged by inaccuracies in antibody homology models. The selection of antibodies for exploring homology model docking simplifies the problem by isolating the various degrees of freedom according to prior knowledge of the uncertainties in an antibody homology model, namely the conformation of the complementarity determining region (CDR) loops (L1, L2, L3 in the light chain, and H1, H2, H3 in the heavy) [4],[5], the hyper-variability of the CDR-H3 loop [6]–[9], and the relative orientation of the antibody light (V_L_) and heavy (V_H_) chains [6], [10]–[12]. A recent study by Narayanan *et al* found that the V_L_-V_H_ relative orientation has a significant impact on the antigen binding properties of an antibody [10], suggesting that simultaneous optimization of the V_L_-V_H_ relative orientation and antibody-antigen relative orientation might capture some of the intramolecular changes undergone by an antibody upon antigen binding.

An additional motivation for studying antibody-antigen complexes is that therapeutic antibodies are revolutionizing healthcare [13]. Oncology, arthritis, immune and inflammatory disorder treatments have benefitted from newly developed therapeutic antibodies [14]. Success of several therapeutic antibody drugs has relied on homology modeling. According to Schwede *et al.*, homology modeling played an important role in the development of 11 of the first 21 marketed antibodies including Zenapax (humanized anti-Tac or daclizumab), Herceptin (humanized anti-HER2 or trastuzumab), and Avastin (humanized anti-VEGF or bevacizumab) [15]. High-resolution computational docking can aid in the design of antibody biologics by providing insights into the complex interactions between an antibody and an antigen [16]. The importance of antibodies combined with the detailed knowledge of the flexibility in the various segments of an antibody fragment variable (F_V_) region make them ideal candidates for the development of novel flexible docking algorithms.

Although there are currently no flexible docking algorithms tailored for antibodies, there have been several efforts to incorporate some of the relevant modes of internal flexibility during docking. Early [17],[18] and more recent approaches [19] use hinges to account for internal flexibility. Multi-body docking, which might be useful for optimizing assembly of V_L_ and V_H_ and antigen chains, has been explored in a few case studies [20],[21] including some targets in the blind prediction challenge known as the Critical Assessment of PRediction of Interactions (CAPRI) [22]. Another genre of docking algorithms like HADDOCK [23] allows flexibility in the side chains and backbones to allow for conformational rearrangements in the interaction surface. Wang *et al.*'s modifications to RosettaDock allow simultaneous gradient-based minimization of the backbone torsional angles and the protein-protein rigid-body orientation [24]. We recently developed a RosettaDock generalization called EnsembleDock [25] that follows the conformer-selection plus induced-fit model [26] to additionally enable docking of a pre-generated ensemble of structures.

We also recently developed RosettaAntibody [27], an antibody F_V_ region homology modeling protocol which incorporates refinement of V_L_-V_H_ relative orientation, CDR H3 loop modeling and minimization of all the CDR loops. RosettaAntibody generates ten antibody homology models for each input sequence, and this set of models can be used simultaneously with EnsembleDock. However, errors in the CDRs of RosettaAntibody homology models (particularly H2 and H3) can still frustrate docking, and only the ten pre-generated backbone conformations are sampled during ensemble docking [27].

In this paper, we discuss the development and implementation of SnugDock, a new antibody docking algorithm built upon RosettaDock using the sampling components of RosettaAntibody. The new protocol performs multi-body docking by allowing simultaneous structural optimization of the relative orientations of antibody-antigen and V_L_-V_H_. SnugDock simulates induced fit by additional simultaneous optimization of the binding interface by allowing flexibility of CDR loops and interfacial side chains. Moreover, we combine SnugDock with EnsembleDock to synthesize a new docking algorithm that encompasses conformer selection, multi-body docking, and a flexible paratope. We test SnugDock using antibody homology models obtained from two accessible public servers, namely RosettaAntibody [28] and Web Antibody Modeling (WAM) [29]. Our goal is to achieve reliable high-resolution antibody docking starting from only the antigen unbound structure and the antibody sequence.

## Results/Discussion

**Figure 1:**
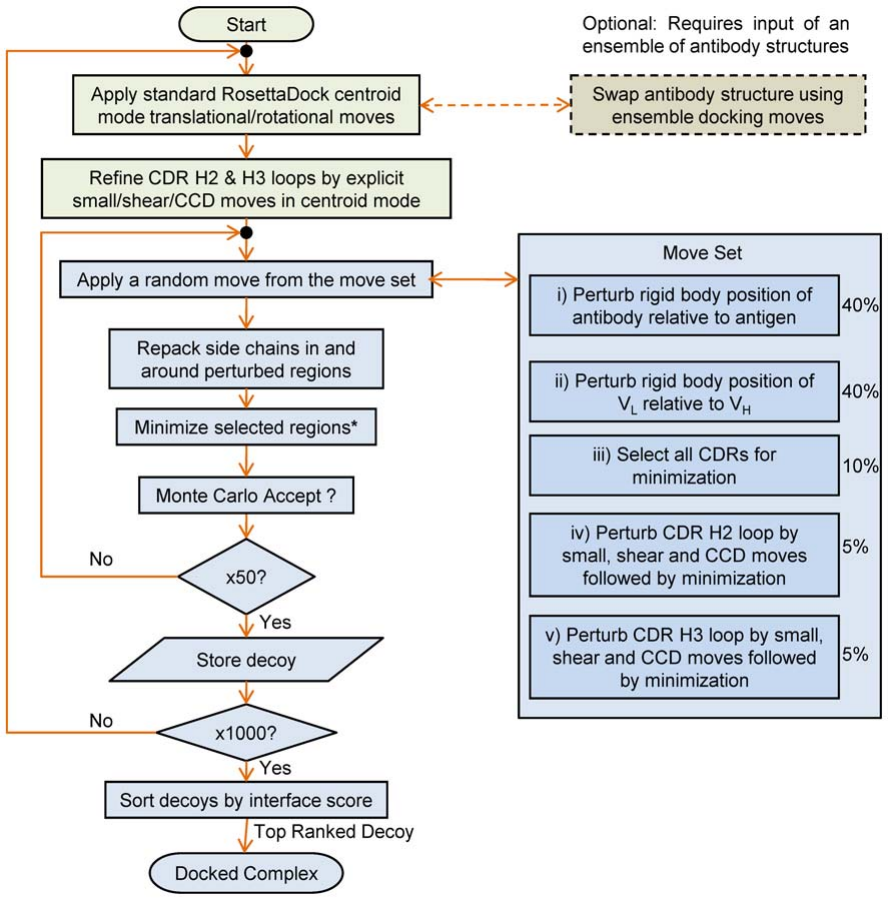
SnugDock flowchart. The low and high resolution stages are shown in shades of green and blue respectively. The Move Set box illustrates five different trial perturbations which are chosen randomly with indicated frequencies. *Rigid body positions are minimized corresponding to the rigid body perturbation move selected. If all the CDRs are selected (see Move Set), they are minimized. If CDRs H3 or H2 are selected for perturbation, they are not subjected to additional minimization since they are already minimized.

Figure 1 summarizes the SnugDock algorithm as incorporated in RosettaDock. Like RosettaDock, SnugDock is divided into a low-resolution and high-resolution stage. In the low-resolution phase (shown in shades of green), SnugDock adds to RosettaDock by additionally perturbing and minimizing the CDR H2 and H3 loops. In each iteration of the high-resolution Monte Carlo-plus-minimization loop (shown in shades of blue), SnugDock randomly chooses a trial move from a set including the various degrees of freedom in both the antibody conformation space and the docking space. Specifically, the trial move set consists of: i,ii) RosettaDock-like rigid body transformations and minimization of either the antibody-antigen or the V_L_-V_H_ orientation; iii) gradient-based minimization of the CDRs L1, L2, L3 and H1 backbones; and iv,v) perturbation and minimization of the backbones of CDRs H2 or H3. The high-resolution iterations also include side-chain rotamer packing steps before each minimization and Monte Carlo Boltzmann acceptance decision (see Methods).

For various tests of the docking algorithm, we input either the crystal structure of the antibody, the lowest-energy (lowest-scoring) RosettaAntibody homology model, the ensemble of the ten lowest-energy RosettaAntibody homology models, or the WAM model. The antibody is docked to the unbound crystal structure of the antigen when available. In the following sections, we first detail the results of a case study as we build up the algorithm, then we summarize the performance of different algorithms on the whole set of antibodies. Next, we delve into the structural details of the sampling. Finally we summarize the results starting from WAM antibody models and the results of global docking.

### Case study: effect of adding internal degrees of freedom in homology model docking

#### Assessment criteria

A docking algorithm can be evaluated by examining plots of a score (an approximation of free energy) versus a measure of distance from the native co-crystal structure for a set of candidate predicted structures. Since protein-protein complexes are presumed to be at equilibrium, the lowest-energy structures generated should match the native structure. Local docking runs, which are typically used to evaluate the ability of an algorithm to refine positions, refine a set of structures near the native complex conformation. In this case, starting positions were created with a local random translation (∼8 Å) and rotation (∼8°) and a spin around the axis of centers of the two proteins (0–360°). Local docking is useful when epitope information is known, as is common in many antibody applications [30]–[32].

**Figure 2:**
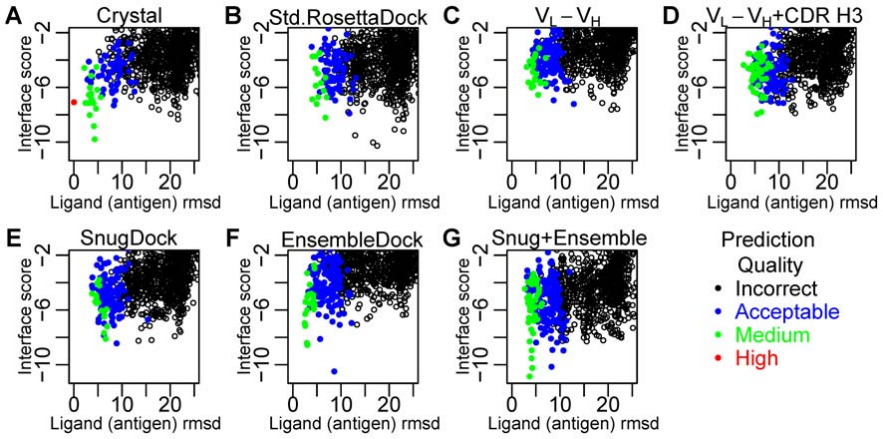
Docking perturbation plots for blocking antibody 11k2 complexed with human monocyte chemoattractant protein (MCP)-1 (2BDN [33]). (A) Standard rigid-body docking using RosettaDock starting with the antibody crystal structure. The red point represents the native crystal structure. (B) Standard rigid-body docking using RosettaDock with the lowest-energy RosettaAntibody model. (C) Docking with V_L_-V_H_ optimization using the lowest-energy RosettaAntibody model. (D) Docking with V_L_-V_H_ optimization with CDR minimization and CDR H3 perturbation using the lowest-energy RosettaAntibody model. (E) Docking with SnugDock (V_L_-V_H_ optimization with CDR minimization and CDR H3+H2 perturbations) using the lowest-energy RosettaAntibody model. (F) Rigid-body docking using EnsembleDock with the ten lowest-energy RosettaAntibody models. (G) Docking using a combined protocol incorporating EnsembleDock and SnugDock with the ten lowest-energy RosettaAntibody models.

Figure 2 shows plots that summarize local docking runs for antibody 11k2 which binds human monocyte chemoattractant protein (MCP)-1 [Protein Data Bank (PDB) ID code 2BDN [33]]. Due to difficulties in accurately capturing the energetic differences from small backbone changes in flexible backbone docking, the interface score (intermolecular energy) provides the best discrimination of structures [24],[25]. For a distance measure, we use the ligand root mean square deviation (rmsd), defined as the rmsd of the antigen N, C_α_, C and O backbone atom coordinates in the predicted structure relative to that in the native crystal structure of the complex after superposition of the antibody N, C_α_, C and O backbone atom coordinates. Candidate structures, or decoys, are rated according to the CAPRI assessment system as high quality, medium quality, acceptable or incorrect based on a combination of the ligand and interface rmsd and the fraction of correct residue-residue contacts (*f*_nat_) across the interface [34]. To test the convergence of a simulation to a correct solution, we define an “energy funnel” to exist when at least five of the ten lowest-energy docking structures are of medium or high quality.

#### Docking with a single antibody homology model

In Figure 2A, docking with standard RosettaDock (fixed backbones and minimization of rotameric side chains [35],[36]) using crystal structures results in the best possible structural prediction of the antibody-antigen complex and serves as the “gold standard” for judging the docking with homology models. The lowest interface-energy decoy is of medium quality, but since there are only four medium quality decoys in the ten lowest interface-energy decoys, a docking energy funnel is not formed.

We wish to compare the docking results when using an antibody homology model. In a blind prediction, RosettaAntibody [27] creates a lowest-energy model of the 11k2 antibody [33] with a 3.1 Å CDR H3 global rmsd-to-native, 1.3 Å rmsd-to-native for all the CDRs, 2.5 Å rmsd-to-native for the relative V_L_-V_H_ orientation, and 1.4 Å rmsd-to-native considering the entire F_V_. Figures 2B–E show sample energy landscapes obtained by docking simulations starting with the lowest-energy RosettaAntibody homology model with the different protocols.

When the lowest-energy RosettaAntibody homology model is docked using standard RosettaDock (Figure 2B), medium and acceptable quality decoys are sampled, but the lowest interface-energy decoy is incorrect, and there is only one medium quality decoy in the ten lowest interface-energy predictions. The poor scores of the medium quality decoys of these native-like structures arise from steric clashes due to the fixed backbone conformation. When clashes are relieved by moving the antigen away from the bound configuration, docking prediction accuracy is lost.

To test the relative importance of the various degrees of freedom in an antibody homology model, we built the SnugDock protocol progressively with increasing degrees of freedom. Figure 2C shows the effect of docking the lowest-energy RosettaAntibody model with a docking algorithm that perturbs and minimizes the relative orientation of both the V_L_-V_H_ and the antibody-antigen pairs. Although the lowest interface-energy decoy is still incorrect, the score difference between the lowest-interface-energy incorrect decoys and lowest-interface-energy acceptable quality decoys has been reduced. There are one medium and two acceptable quality decoys in the ten lowest interface-energy decoys. The interface scores produced by this run are higher than those in the standard RosettaDock run. The additional degrees of freedom allow lower total scores to be accessed, but those lower scores are achieved at the expense of the interface. That is, V_H_-V_L_ interactions and some intra-chain energies are improved, but the Ab-Ag interaction suffers. Additional types of sampling are necessary to recover a low-energy interface.

Following the incorporation of the V_L_-V_H_ optimization, we added CDR minimization along with explicit perturbations to the CDR H3 loop conformation (Figure 2D). The lowest interface-energy decoy is now a medium quality prediction, but since there are only two medium quality predictions in the ten lowest interface-energy decoys, an energy funnel is still absent.

Based on findings in our previous work in antibody-antigen binding [27], that in some cases CDR H2 played a key role and had the highest deviation from crystal structures amongst the grafted CDRs, in the final step we added explicit perturbations to the CDR H2 loop. The explicit perturbation of the CDR loops H2 and H3 combined with the minimization of all CDR loops, rigid body optimization of the relative V_L_-V_H_ orientation, and simultaneous docking of the antigen results in the synthesis of a docking algorithm with complete paratope optimization. With the full set of antibody degrees of freedom, we call this implementation SnugDock. With SnugDock the lowest interface-energy structure is of acceptable quality (Figure 2E). This structure has a high ligand rmsd of 8.9 Å and an interface rmsd of 4.2 Å but still meets the CAPRI acceptable criteria because the fraction of native residue-residue contacts (*f*_nat_) is 39%, greater than the threshold of 30%. The fourth decoy is of medium quality, with ligand rmsd of 6.6 Å, interface rmsd of 3.5 Å and *f*_nat_ of 55%, surpassing the stringent cutoff of 50%. There is one additional medium and acceptable quality prediction in the ten lowest interface-energy decoys.

#### Ensemble methods

Our previous work demonstrated that using EnsembleDock with ten RosettaAntibody homology F_V_ models results in more accurate docking predictions than possible by standard RosettaDock [27]. For a docking run with EnsembleDock, the lowest interface-energy decoy is acceptable quality (Figure 2F). In the ten lowest interface-energy decoys there are four medium quality predictions.

Finally, we have combined SnugDock and EnsembleDock by using all of the previous antibody sampling steps in addition to a conformer-selection step that samples from ten pre-generated RosettaAntibody models (Figure 2G). With the combined algorithm, the lowest interface-energy decoy is of medium accuracy and is better than the lowest interface-energy decoys of both the independent SnugDock (Figure 2E) and EnsembleDock (Figure 2F) simulations. The EnsembleDock-plus-SnugDock approach results in five medium quality decoys in the ten lowest interface-energy decoys, giving rise to a docking energy funnel and suggesting that the combined algorithm is more robust. The lowest interface-energy decoy generated by the combined protocol scores lower than the lowest-energy decoy of the independent simulations. The synergy demonstrated by the combined algorithm arises from EnsembleDock sampling the backbone space more broadly and SnugDock refining the antibody-antigen interface.

#### Benchmark set comparison of algorithms

**Figure 3:**
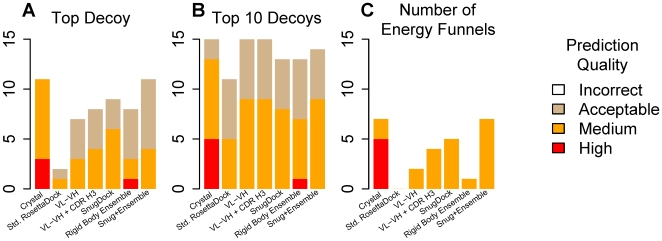
Summary of docking performance. The bar plots show the number of correctly docked targets out of fifteen targets for different docking algorithms. (A) Docking performance considering the lowest-energy decoy. (B) Docking performance considering the most native-like prediction in the ten lowest-energy decoys. (C) Docking performance based on the presence of docking energy funnels. *Crystal* indicates standard rigid-body docking using crystal structures. *RosettaAntibody* indicates standard rigid-body docking using RosettaDock starting with the lowest-energy RosettaAntibody homology model. *VL-VH* indicates docking with V_L_-V_H_ optimization. *VL-VH+CDR H3* indicates docking with V_L_-V_H_ optimization with CDR minimization and CDR H3 perturbation. *SnugDock* indicates docking using SnugDock. *Rigid Body Ensemble* indicates rigid-body docking using EnsembleDock with the ten lowest-energy RosettaAntibody homology models. *Snug+Ensemble* indicates docking using the EnsembleDock-plus-SnugDock combined protocol with the ten lowest-energy RosettaAntibody homology models.

While the 2BDN case demonstrates the type of analysis we use to evaluate differing algorithms, it is necessary to consider a broader set of targets to draw general conclusions. We identified 15 antibodies with known complex structures and H3 loop lengths between 7 and 11 residues, the range where loop structure prediction is of medium accuracy [27],[37]. The full set of local docking plots for all algorithm variants and all fifteen targets is in Supporting Information Figure S1. Table 1 shows the CAPRI rating of the lowest interface-energy model for each of the fifteen targets and presents the three metrics of docking accuracy as each additional degree of freedom is incorporated into the algorithm. The statistics are summarized in a histogram in Figure 3.

**Table 1:** Accuracy of lowest-energy docking decoy for different docking protocols.

			Crystal	RosettaAntibody Homology Model					
Co-Crystal PDB ID	Type (Ab-Ag) U = Unbound B = Bound	CDR H3 Length	Standard RosettaDock	Standard RosettaDock	Optimize V_L_-V_H_ RosettaDock	Optimize V_L_-V_H_ Minimize CDRs Relax H3 RosettaDock	Optimize V_L_-V_H_ Minimize CDRs Relax H3+H2 RosettaDock (SnugDock)	Rigid Body Ensemble	EnsembleDock-plus-SnugDock
1mlc	U(1mlb)-U(1lza)	7	0	0	0	0	**	0	*
1ahw	U(1fgn)-U(1boy)	8	**f	0	*	*	**f	*	**f
1jps	U(1jpt)-U(1tfh)	8	**f	*	*	*f	**f	**	*f
1wej	U(1qbl)-U(1hrc)	8	**	0	*	*	*	*	*
1vfb	U(1vfa)-U(8lyz)	8	0	0	0	0	0	0	0
1bql	B-U(1dkj)	7	**	0	**f	**f	**f	**	**f
1k4c	B-U(1jvm)	9	**	0	0	**	0	*	**f
2jel	B-U(1poh)	9	0	0	*	0	*	*	*
1jhl	B-U(1ghl)	9	0	0	0	0	0	0	0
1nca	B-U(7nn9)	11	**f	0	0	0	0	0	0
2bdn	B-B	8	**	0	0	**	*	*	**f
1ynt	B-B	9	**f	**	**f	**f	**f	0f	*f
2aep	B-B	9	***f	0	**	*f	**f	0	*
2b2x	B-B	10	***f	0	0	0	0	0	0f
1ztx	B-B	10	***f	0	0	0	0	***	*
	CAPRI Summary for Top Decoy		3***/8**	1**/1*	3**/4*	4**/4*	6**/3*	1***/2**/5*	4**/7*
	CAPRI Summary for Top 10 Decoys		5***/8**/2*	5**/6*	9**/6*	9**/6*	8**/5*	1***/6**/6*	9**/5*
	Number of Funnels		7	0	2	4	5	1	7
	Time Per Decoy (min)		6	6	8	14	17	7	18
	Time Per Result (CPU-hours)		100	100	133	233	280	120	300
	Relative Compute Time		1.0	1	1.3	2.3	2.8	1.2	3.0

All simulations were carried out using the lowest-energy RosettaAntibody homology model, except (1) the *Crystal* column where the crystal structure of the antibody was used, and (2) the last two columns where the ensemble of the ten lowest-energy RosettaAntibody homology models were used as EnsembleDock starting structures. *Type* indicates bound (B) or unbound (U) starting structures for docking for the antibody (Ab) or antigen (Ag). The corresponding PDB IDs of the unbound structures are noted in parentheses. The number of residues in the CDR H3 loop is provided since it is at the center of the paratope and is a key contributor to accurate antibody-antigen interactions. Docking performance is indicated using CAPRI ratings [34]: Three stars (***), two stars (**), one star (*) and “0” indicate that lowest-energy docking decoy is of high, medium, acceptable and incorrect quality, respectively. Predictions marked “f” additionally have a docking energy funnel defined where five of the ten lowest-energy structures are of medium or high quality. CAPRI summary lines show the total number of targets for which the lowest-energy docking decoy and the most accurate model in the ten lowest-energy docking decoys is of high/medium/acceptable quality. Full quantitative measurements underlying the CAPRI ratings, of the predicted models are available in Supporting Information Table S1.

The gold standard of docking crystal structures with standard RosettaDock results in three and eight targets with lowest interface-energy structures of high and medium quality, respectively, and seven targets with funnels. However, when antibody homology models are used as inputs to standard RosettaDock, the successes fall to one medium and one acceptable quality result and no funnels. As the degrees of freedom are added towards building the SnugDock protocol, the number of successful predictions increases. Homology model docking using the combination EnsembleDock-plus-SnugDock protocol has similar number of targets with acceptable or better predictions as the use of crystal docking with standard RosettaDock, but the quality of docking predictions is still much better for simulations starting with the antibody crystal structure. Evaluation of docking protocols by a looser criterion involving analysis of the most native-like decoy in the ten lowest interface-energy predictions (Table 1; Figure 3B) shows that, irrespective of the chosen protocol, a prediction of at least an acceptable quality is obtained for most targets. The number of docking funnels produced by each protocol also increases steadily with the incorporation of additional degrees of freedom (Table 1; Figure 3C), indicating that for local docking with homology models any flexible docking protocol is better than standard rigid-body docking.

In general, the results demonstrate that targeted flexibility in the antibody can overcome the inaccuracies inherent in a homology model and result in higher docking accuracy. The five energy funnels produced by the full SnugDock algorithm and the seven funnels produced by the EnsembleDock-plus-SnugDock protocol suggest more confident and robust predictions since more low-interface-energy decoys are native-like. Despite the general trends, results vary by individual target. For example, for 2BDN or 2B2X, accuracy improves as more degrees of freedom are used. But in other targets, such as 1MLC or 2AEP, some steps result in decreased performance.

The improvements in the prediction accuracy of antibody-antigen complex structures are achieved at a computational cost. The penultimate row of Table 1 shows the average total time required by a single-core 2.33 GHz CPU to generate a result for one target. The last row of Table 1 shows the effective simulation time, relative to standard RosettaDock, required for each algorithm to create a single candidate structure. The full EnsembleDock-plus-SnugDock protocol requires 300 CPU-hours per prediction, roughly three times more expensive than standard rigid-body RosettaDock.

### High-resolution analysis of SnugDock decoys

#### Structural diversity generated by SnugDock

**Figure 4:**
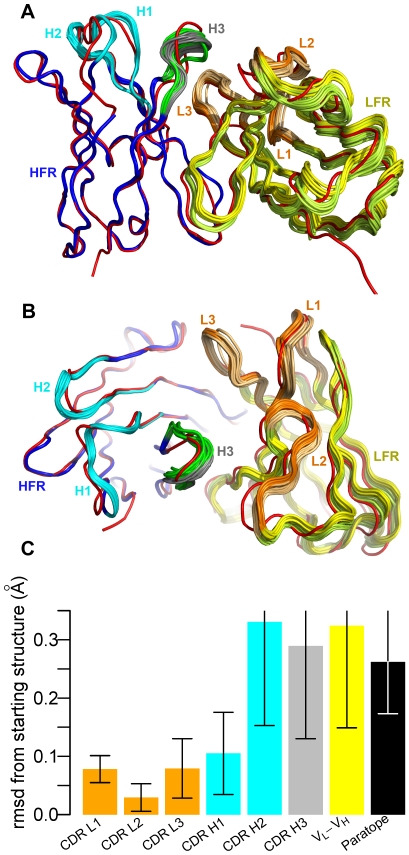
SnugDock conformational diversity. (A) The diversity in conformation generated by SnugDock during docking of anti-HEL Fab fragment (1BQL) to bobwhite quail lysozyme (1DKJ). (B) View facing the paratope. Crystal structure, red; heavy and the light chains, blue and yellow, respectively; light and heavy chain CDRs, orange and cyan, respectively; SnugDock sampled CDR H3, grey; EnsembleDock-plus-SnugDock sampled CDR H3, light chain CDRs and light chain framework, green, light orange and yellow-green, respectively. Structures are all superposed onto the heavy chain framework residues of the crystal structure. (C) Mean rmsd from the starting structure of the ten lowest-energy docking decoys for fifteen targets. For light and heavy chain CDRs, the corresponding framework chain is superposed and the rmsd is queried over the respective CDR residues. V_L_-V_H_ denotes the rigid-body rmsd divergence of the heavy chain framework when the light chain framework is superposed. The paratope comprises all CDRs, and the rmsd was computed by superposing the paratope and querying over the same residues. The colors of the bar correspond to the colors of the different antibody segments in (A) and (B). The error bars denote one standard deviation.

A major objective of this study was to generate structural diversity in the backbone to compensate for errors in the antibody homology model. Figures 4A–B show side and top views of a set of models for a representative target to show the structural diversity generated by SnugDock or the EnsembleDock-SnugDock combination. The diversity of the light chain framework (yellow) arises from the SnugDock V_L_-V_H_ rigid-body perturbation. With SnugDock alone, the CDR loops (other than H2 and H3) have small variations from the minimization steps. The structural diversity of the CDR H3 (grey) and H2 (cyan) is significantly broader, reflecting the additional sampling by explicit φ-ψ perturbations. Note the discrepancy between the native CDR H3 conformation (red) and the models (grey). With EnsembleDock-plus-SnugDock, CDR H3 structures (green) spread more broadly, thus the combined algorithm samples more diverse conformations than possible by SnugDock alone. The gap between the native CDR H3 conformation and the SnugDock generated CDR H3 conformations is partially bridged by the EnsembleDock-plus-SnugDock generated CDR H3 conformations.

The generated diversity is summarized quantitatively in Figure 4C. CDRs that have been subjected to minimization only, *viz.* CDRs L1, L2, L3 and H1, exhibit a mean divergence of less than 0.1 Å rmsd from the starting structure. CDRs H2 and H3, which are subjected to explicit perturbation, sample a larger conformational space and show a mean fluctuation of about 0.3 Å rmsd from the starting structure. The relative V_L_-V_H_ orientation, which is subjected to rigid body moves followed by minimization, also exhibits a similar divergence. The paratope as a whole, influenced by both the loop conformations and the relative orientation of the heavy and the light chains, has a mean rmsd of 0.3 Å to the starting structure. These deviations enable the antibody to sample lower energy conformations, but are not typically large enough to capture the full transition from the homology model to the bound conformation. Homology modeled CDR H3s, for example are typically 1–3 Å away from the bound conformation, and this range is similar to the diversity of conformations of low-energy antibody models used in EnsembleDock.

#### Successes: paratope optimization can help recover native-like decoys

**Figure 5:**
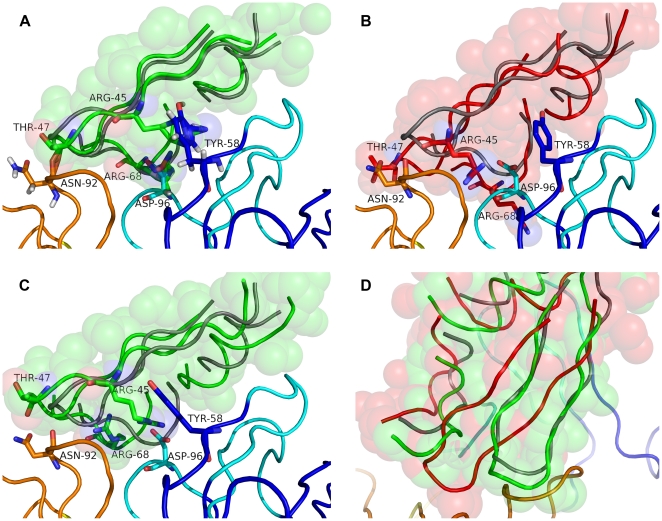
Structural details of the monoclonal antibody Fab D44.1 complexed with lysozyme (1MLC [38]). (A) The interface region of the lowest-energy RosettaAntibody homology model for target 1MLB complexed with the crystal structure of lysozyme (1LZA). (B) The interface region of the most native-like prediction in the ten lowest-energy docking predictions on docking with standard rigid-body RosettaDock. (C) The interface region of the most native-like prediction in the ten lowest-energy docking predictions on docking with SnugDock. (D) Superposition of the structures shown in (B) and (C) viewed facing the binding region from the antigen's side. Conformations of the antigen in the crystal structure, green; predicted by standard rigid-body RosettaDock, red; and that predicted by SnugDock, grey; heavy and light chains, shades of blue and yellow respectively. Sticks indicate the labeled residues that have relieved the steric clash present in the starting structure due to the flexibility allowed by SnugDock. Transparent spheres indicate the interface region of the predicted conformation of the antigen. The light and heavy chain frameworks of the predicted complexes are superposed on the corresponding residues of the antibody in the crystal structure.

Figure 5 shows the interface of the complex structure formed by Fab D44.1 and lysozyme (1MLC [38]). Aligning the lowest-energy RosettaAntibody F_V_ homology model with the bound crystal conformation of the antibody in the crystal complex gives rise to clashes with the bound conformation of the antigen (Figure 5A). Specifically, antigen residues Arg-45, Thr-47 and Arg-68 clash with antibody residues Tyr-58H (in CDR H2), Asn-92L (L3) and Asp-96H (H3) respectively. The clashes arise from the structural deviation in the loops: global rmsd-to-native of CDRs L3, H2 and H3 of the F_V_ homology model is 0.7 Å, 1.0 Å and 1.9 Å respectively. After docking with standard RosettaDock, the most native-like decoy in the ten lowest interface-energy docking solutions does not have any clashes (Figure 5B), but the antigen (red) is displaced from its bound orientation (green) resulting in an acceptable quality model. After the paratope is altered by SnugDock, the CDRs H3 and L3 have similar global rmsd, and CDR H2 moves slightly closer to the native. SnugDock relieves the clashes while keeping the antigen (grey) close to the bound conformation (green), resulting in a medium quality model (Figure 5C). The paratope, comprising all the CDR loops in the SnugDock generated model (Figure 5C), is 0.1 Å rmsd closer to the bound crystal conformation than that of the starting structure. A superposition of the structures predicted by standard RosettaDock and SnugDock (Figure 5D) shows that the antigen orientation predicted by standard RosettaDock is rotated by ∼25° compared to the crystal structure, whereas the antigen orientation predicted by SnugDock is very close to the native orientation. This example is typical of those for which SnugDock improves docking. SnugDock allows the antibody to find a way to fit without clashes, but does not necessarily move the antibody's internal conformation closer to its native bound backbone structure.

#### Failures: dangers of over-optimizing the binding interface

**Figure 6:**
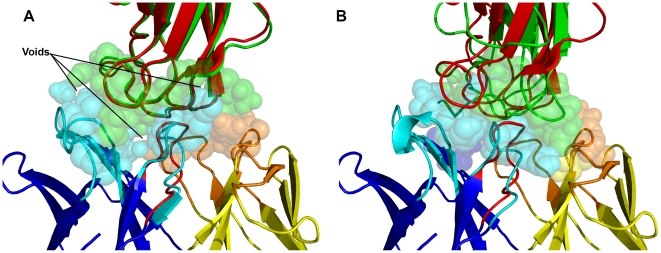
Predicted models of the complex of west Nile virus envelope protein DIII with neutralizing E16 antibody Fab (1ZTX [39]). (A) Lowest-energy prediction (medium accuracy) generated by EnsembleDock-plus-SnugDock simulations ranked by all-atom score of the entire complex. (B) Lowest-energy prediction (acceptable accuracy) generated by EnsembleDock-plus-SnugDock simulations ranked by the intermolecular components of the all-atom score. The light (deep blue) and heavy (yellow) chain framework of the docked antibody is superposed on the corresponding residues of the crystal complex. Predicted orientation of the antigen, green; light and heavy chain CDRs, orange and cyan respectively; CDR H3 loop and antigen in the crystal structure, red; residues at the interface, transparent spheres.

The docking accuracy of EnsembleDock-plus-SnugDock is typically equal or better than using EnsembleDock alone. One exception is the complex of west Nile virus envelope protein DIII with neutralizing E16 antibody Fab (1ZTX [39]). EnsembleDock produces a high-quality top-ranked structure whereas EnsembleDock-plus-SnugDock produces only an acceptable accuracy structure. Interestingly, if the EnsembleDock-plus-SnugDock decoys for target 1ZTX are sorted by the score of the entire complex instead of the interface score, the lowest energy decoy is of medium quality and a docking funnel is formed. The lowest total-energy decoy has a CDR H3 global rmsd of 1.7 Å, whereas the lowest interface-energy decoy has a CDR H3 global rmsd of 2.7 Å. The 1.7 Å CDR H3 loop model has a poorly packed interface with voids, penalizing the interface score (Figure 6A). Surprisingly, the 2.7 Å CDR H3 model shows a more compact interface (Figure 6B) resulting in a better interface score. In this case, the native crystal structure shows interfacial water molecules which are poorly captured by Rosetta's implicit solvation model.

A second failure case is the complex between neuraminidase from influenza virus and the monoclonal antibody NC41 (1NCA [40]). Docking with crystal structures produced a docking energy funnel, however, none of the other methods were capable of producing even an acceptable prediction in the ten lowest-interface-energy decoys. The failure may be due to loop modeling errors in regions critical for binding. The 1NCA CDR H3 loop prediction is one of the poorest ones in our dataset: the lowest-total-energy RosettaAntibody homology model has 3.4 Å global rmsd, and even the most native-like CDR H3 loop in the ten lowest-total-energy homology models has a global rmsd of 2.2 Å. 1NCA has the longest H3 loop in our dataset (11 residues), and the H3 loop makes key contacts with the antigen. Notably, other cases with poor loop models dock successfully (e.g. 1VFB, 2B2X), but typically those cases have interfaces which do not involve as many CDR H3 contacts.

### SnugDock applied to WAM antibody homology models

A longstanding source of homology models is the Web Antibody Modeling server (WAM) created by Whitelegg and Rees [29]. The WAM server grafts antibody components together and models the H3 loop *de novo*. SnugDock may be able to compensate for the model errors during docking. Ensemble docking is not possible since WAM returns only one model for a given sequence. Table 2 presents the accuracy of docking predictions obtained by using WAM antibody homology models using both standard RosettaDock and using SnugDock. The lowest-interface-energy docking decoy generated by standard rigid-body docking simulations using WAM homology models are almost all incorrect. Subjecting the WAM models to SnugDock resulted in six medium quality lowest-interface-energy docking decoys. Thus SnugDock recovers more accurate docking predictions. The original WAM homology models showed strain in the molecule reflected in high Rosetta scores. By subjecting the homology model to minimization on the paratope degrees of freedom, SnugDock was able to relieve intramolecular and inter-chain steric clashes. Interestingly, the SnugDock results with WAM models are comparable to those with RosettaAntibody models, while the use of EnsembleDock-plus-SnugDock with the RosettaAntibody models achieves higher accuracy.

**Table 2:** Accuracy of lowest-energy docking decoy, using WAM homology models.

Co-Crystal PDB ID	Standard RosettaDock	SnugDock
1mlc	0	**f
1ahw	**f	*
1jps	*	**
1wej	0	**
1vfb	0	**f
1bql	0	*
1k4c	0	*
2jel	0	0
1jhl	0	0
1nca	0	0
2bdn	0	*
1ynt	**	**
2aep	0	**f
2b2x	*	*
1ztx	0	0
CAPRI Summary for Top Decoy	2**/2*	6**/5*
CAPRI Summary for Top 10 Decoys	4**/7*	1***/9**/4*
Number of Funnels	1	3

Refer to Table 1 key for explanation. Full quantitative measurements underlying the CAPRI ratings, of the predicted models are available in Supporting Information Table S2.

### Global docking

Local docking is often of interest in antibody applications since epitope information can be obtained by a variety of other methods. However, global docking is a computational alternative for producing epitope information when it is unknown. Global docking can be significantly more challenging because of the larger conformational space to search. Further, flexible docking creates additional danger of creating an unrealistic induced fit at a non-native docking location, resulting in a false positive prediction [41],[42]. Global docking is considerably more computationally demanding, and thus we restricted our tests of global docking to five targets, and to simulate a practical docking application, chose only those targets for which unbound crystal structures were available for both the antibody and the antigen: 1MLC, 1AHW, 1JPS, 1WEJ and 1VFB. The starting structures consisted of the unbound crystal structure of the antigen and the lowest-interface-energy RosettaAntibody homology model. The EnsembleDock and the EnsembleDock-plus-SnugDock protocols used the ten lowest-interface-energy RosettaAntibody homology models. For each target, we generated 5000 candidate structures, with each prediction run beginning from a random global rotation of the antigen and a small perturbation of the antibody (to keep the paratope generally directed toward the antigen).

Global docking using standard rigid-body RosettaDock generated no acceptable quality lowest-interface-energy decoys for any targets, and a top-ten ranked acceptable decoy for only one target (Table 3). Using EnsembleDock or SnugDock independently produced marginal improvement with a few acceptable quality predictions. The combination algorithm of EnsembleDock-plus-SnugDock generated two medium quality lowest-interface-energy predictions exhibiting the synergy already established in the local docking simulations. Additionally, the most native-like decoy in the ten lowest-interface-energy decoys was of medium quality for three of the five targets and acceptable for one target. The results are comparable to global docking using standard rigid-body RosettaDock with unbound crystal starting structures of the antibodies, where one structure had a high quality (1JPS), two had medium quality, and the others had acceptable quality prediction for the most native-like decoy in the ten lowest-interface-energy decoys. The results can also be compared with local docking using EnsembleDock-plus-SnugDock around the known epitope (Table 1), where four of the five targets had at least a medium quality prediction for the most native like decoy in the ten lowest-interface-energy decoys, and the fifth target (1VFB) had an acceptable prediction. Thus one target (1VFB) which had succeeded in local docking failed in global docking due to low-scoring non-native decoys (see docking energy landscapes, Supporting Information Figure S2). In general, addition of SnugDock increases sampling of more native-like decoys, enabling near-natives to be energetically more favorable.

**Table 3:** Accuracy of global docking decoys.

	Unbound Crystal Structures		RosettaAntibody Homology Model							
	Standard RosettaDock		Standard RosettaDock		EnsembleDock		SnugDock		EnsembleDock-plus-SnugDock	
Co-Crystal PDB ID	Top Decoy	Top 10 Decoys	Top Decoy	Top 10 Decoys	Top Decoy	Top 10 Decoys	Top Decoy	Top 10 Decoys	Top Decoy	Top 10 Decoys
1mlc	0	**	0	0	0	0	0	0	0	*
1ahw	**	**	0	0	*	*	*	*	**	**
1jps	**	***	0	*	0	*	0	*	**	**
1wej	0	*	0	0	0	0	*	*	0	**
1vfb	0	*	0	0	0	0	0	0	0	0

Refer to Table 1 key for explanation. Full quantitative measurements underlying the CAPRI ratings, of the predicted models are available in Supporting Information Table S3.

### Discussion

SnugDock is the first docking algorithm with targeted antibody flexibility. SnugDock models flexible loop conformations, backbone motions, and inter-chain (V_H_-V_L_) adjustments. The introduction of flexibility during docking is critical to overcome the inaccuracies inherent in a homology modeled antibody structure. Comparison of algorithms shows that increasing the degrees of freedom in local docking gradually increases the quality of predictions. Ultimately, EnsembleDock-plus-SnugDock with homology models achieves accuracy comparable to docking crystal structures with standard RosettaDock. While the algorithm is limited to antibody-antigen interactions, the results suggest that it is possible for computational predictions to use homology models to bridge the gap between the number of experimentally determined complex structures and the available sequences of pairs of interacting proteins.

In CAPRI rounds 1–18, eleven of the forty targets involved docking of at least one homology modeled partner (both partners were homology models for Target 35) [43]–[45]. For six of the eleven targets, none of the participating groups could predict any medium or higher accuracy structures. When the sequence identity was under 40%, the solutions were of acceptable quality at best (Targets 20, 24). High quality predictions were obtained only for two cases (Targets 14, 19) and in both cases the binding region of the template structure was structurally similar to the co-crystal structure [44], and the other docking partner was in the bound conformation. The poor performance of homology modeled docking partners in CAPRI highlights the need of docking algorithms like SnugDock which are robust to inaccuracies in a homology model. Targets 20 and 24 with only acceptable predictions had poorly modeled loop and C-terminal regions which were responsible for key contacts in the native binding interface [45], showing that using homology models with loops at the binding region makes docking with homology models even harder. SnugDock with its loop relaxations at the binding interface addresses the challenge toward accurate high-resolution predictions.

The CDR H3 loop of antibodies provides the most diversity and is thus a focus of the conformational sampling in the SnugDock algorithm. In our experience with the RosettaAntibody Server [28], there are antibodies with non-H3 loops which elude the traditional Chothia classification system [4] and may not fit into canonical CDR templates. The SnugDock algorithm is easily generalizable to include perturbations of loop conformations for any of the six CDR loops. Extra sampling however should be restricted to special cases for efficiency and to avoid issues with over-optimized, non-native induced-fit structures. For approaching non-antibody targets, the SnugDock methods would need to be adapted requiring knowledge of a binding site and appropriate choices of loops to target flexibility. The multi-chain docking methods can be applied to any multi-chain docking partner.

The flexible docking methods help in identifying the correct docked complex structure, but unfortunately they do not yet help in refining the monomer homology structures themselves closer to the crystal backbone conformations. This limitation likely arises from the vast conformational space of the backbone and the difficulties with high-resolution refinement of protein structures [46],[47]. In docking, some of the energetic issues are avoided through the use of the interface energy instead of the total energy. Further advancements in refinement techniques will be needed to address this shortcoming. SnugDock's advancements in the sampling problem also reveal continuing issues with the knowledge of nature's energy function. Missing water molecules affected the prediction of targets 1ZTX and 1VFB. Antibody interfaces in general are polar [48], and several targets with the most polar interfacial CDRs (1VFB, 1JHL, 1NCA) failed perhaps due to the challenges in modeling electrostatics [49].

Experimental techniques for epitope mapping like hydrogen-deuterium mass spectroscopy [50] can help to pre-orient an antigen for local docking. However, when such data are not available, one must resort to global docking where the docking simulations are started with random orientations of the docking partners. Our limited testing of global docking encouragingly suggests that the EnsembleDock-plus-SnugDock approach can successfully find epitopes. Global searches should still be performed with care as the large conformation space can frustrate the ability to find the native binding interface or obscure it through false positive non-native interaction.

One could envision a complete computational antibody engineering pipeline starting from the antibody sequence and ending with accurate predictions for optimized antibody-antigen interactions. In this paper we have been successful in reaching the second step by computational docking using computational models of the monomer antibody. The next stages may be additionally challenging. High-resolution complex structures might next be used for computational alanine scanning [51], computational affinity maturation [52] or alteration of binding specificity [53]. For antibody therapeutics, structures will help define drug mechanisms for regulatory approval [15], enable epitope mapping [54] and humanize constructs [55]. These applications require varying amounts of resolution and further testing will reveal the full utility of the SnugDock predictions.

## Methods

### Antibody-antigen benchmark

To compare with prior work, we use the set of fifteen antibody-antigen complexes as tested in the original RosettaAntibody publication [27]. The antibody-antigen complex dataset was compiled to ensure: 1) a fair representation of unbound-unbound, unbound-bound and bound-bound antibody-antigen docking targets, 2) a spectrum of CDR H3 loop lengths (7 to 11 residues) and 3) both old and newly released crystal complexes (PDB release dates 1992–2006). The RosettaAntibody and the WAM structures are as reported previously [27].

### SnugDock Protocol

SnugDock is implemented in the Rosetta biomolecular modeling suite. Fold trees objects [24] are used to guide the propagation of structural changes during docking with backbone flexibility. One fold tree uses flexible jumps for moving the V_L_-V_H_ and antibody-antigen pairs relative to each other. A second fold tree for CDR loop relaxation had fixed jumps joining the loop stems, and cuts at the middle of the loops. Move map objects are used to select particular sets of residues for backbone and/or side-chain torsion angle flexibility.

Figure 1 depicts the flowchart for the steps in the SnugDock protocol, implemented as follows. Steps 1 and 2 describe the initial setup, Steps 3–6 describe the low resolution stage and steps 7–13 describe the high resolution stage.

1. Orient the antigen randomly:
  - Local perturbations: From a superposition of the antibody in the bound orientation, spin antigen randomly around the axis connecting the center of masses of the antibody and the antigen, tilt (8°) away from the same axis and translate (8 Å), similar to earlier treatments [35].
  - Global perturbations: Randomly orient the antigen without using any information of the antigen's orientation in the crystal structure. Point antibody paratope towards antigen.
2. Slide into glancing contact as defined by at least one antibody-antigen atomic contact within 1 Å of van der Waals contact distances.
3. Perturb the antibody by rigid-body transformations following the low resolution phase in RosettaDock [35].
4. Optional for input comprising of an ensemble of antibody structures: Select a structure from the ensemble of input antibody structures by Monte Carlo swapping as described previously [25].
5. Repeat steps 3 and 4, fifty times.
6. Optimize the CDR H2 & H3 loop conformations by small [56], shear [56], and cyclic coordinate descent (CCD) [57] moves and gradient-based minimization in low resolution as detailed previously [27] except with the side chains represented as one pseudo-atom (no side-chain packing). The fold tree [27] is modified here to prevent the relative V_L_-V_H_ movement at coarse resolution.
7. Change representation of the protein from the low resolution to full atomistic detail by using the side-chain conformations from the starting antibody homology model and the unbound antigen crystal. If the unbound crystal structure of the antigen is not available, the antigen is packed with side-chains from a rotamer library.
8. Optimize the side-chain conformations of the residues at the V_L_-V_H_ interface, antibody-antigen interface and all CDR loops and neighboring residues (within 8 Å) by rotamer packing and minimization including the unbound side chain conformations as described previously [36],[58].
9. Choose and apply a move from the move set. The move set consists of five kinds of moves (the probability of each move is indicated in percentages):
  - Rigid body perturbation of the antigen relative to the antibody using the parameters from rigid-body perturbation in the standard RosettaDock algorithm. (40%)
  - Rigid body perturbation of the relative V_L_-V_H_ orientation using the parameters from rigid-body perturbation in the standard RosettaDock algorithm. (40%)
  - Minimization of the backbone residues of all the CDR loops. (10%)
  - Relaxation of CDR H3 by small, shear and CCD moves followed by minimization as detailed previously [27] with the fold tree modified to prevent the relative V_L_-V_H_ movement. (5%)
  - Relaxation of CDR H2 by small, shear and CCD moves followed by minimization as in Step 9iv. (5%)
10. Optimize selected side-chains as described in Step 8: For rigid body perturbations (following moves 9i and 9ii), the relevant interfacial side chains are selected for optimization. For loop optimizations (following move 9iii), side chains of the loop and neighboring residues are selected for optimization.
11. Minimize selected region: Rigid body positions (following move 9i or 9ii) or all the CDRs (following move 9iii). CDRs H3 (move 9iv) or H2 (move 9v) are not subjected to additional minimization.
12. Steps 9–11 are repeated fifty times and each iteration is accepted or rejected based on a Monte Carlo criterion (temperature, kT = 0.8).
13. The lowest interface-energy structure observed during the course of the simulation is selected as the output of the simulation.

Each decoy of an independent docking simulation begins from a different random starting position. In local docking, the set of starting positions comprises a diffuse cloud that covers a reasonable area (∼20 Å rmsd) with moderate density around the native ligand position. In local and global docking, 1,000 and 5,000 candidate docking structures are generated for each target respectively. In our empirical tests, 5,000 decoys were sufficient and results were similar for test runs of 10,000 decoys in a subset of targets. The energy function used during the course of the simulation is as described previously [35] with (i) the interfacial terms of the scoring function including both the antibody-antigen interface and the V_L_-V_H_ interface, and (ii) chain break penalties for six CDR loops. Interface energy [24] is used to rank and discriminate the structures produced by the docking simulations.

#### Variation in methods

Two algorithm variations we tested require brief mention. First, incorporation of CDR H2 and H3 relaxation in low resolution generated a threefold increase in the diversity of loop conformations than that generated by the relaxations in full atom representation of the high resolution alone, and improved docking accuracy. Second, early attempts at simultaneous gradient-based minimization over all degrees of freedom (antibody-antigen, V_L_-V_H_, non H2 & non H3 CDRs, CDR H2, CDR H3) resulted in only small perturbations to the relative orientation of the antibody and the antigen, while all the other degrees of freedom remained unaltered. Better sampling was achieved by isolating the various degrees of freedom and carrying out multiple rounds of minimization over one randomly selected degree of freedom while fixing the other degrees. Isolating the degrees of freedom increased computational efficiency, and required about one-third the time required for simultaneous minimization.

### Docking Metrics

Ligand rmsd is the deviation of the N, C_α_, C and O backbone atoms of the antigen after superposition of the antibody backbone atoms. Interface rmsd is the deviation of the backbone atoms at the interface after optimal superposition of those same atoms, where the interface is defined as all residues within 10 Å of a non-hydrogen atom of the other docking partner. Interface energy is the component of the total docking score that arises from inter-molecular residue-residue interactions at the antibody-antigen interface. For *f_nat_* calculations, residue-residue contacts are defined when a residue is within 5 Å of a non-hydrogen atom from the other docking partner. The docking models are assigned CAPRI [22]-style “high”, “medium”, “acceptable” or “incorrect” rankings that depend on the rmsd-to-native of the ligand position, the interface rmsd to native and the fraction of native residue-residue contacts (*f*_nat_) that are recovered in the docked model [59]. Convergence of a docking simulation is indicated by the presence of a docking funnel, which is defined to exist if there are at least five medium quality predictions in the ten lowest-energy docking decoys.

### Algorithm availability

The SnugDock method presented here is freely available for academic and non-profit use as part of the Rosetta structure prediction suite at www.rosettacommons.org. The distribution includes documentation and full source code. The Rosetta version numbers and command lines used to generate the data are provided in Supporting Information Text S1.

## Supporting Information

Figure S1 Docking perturbation plots. Each row shows the simulation for one target denoted by the four letter PDB code at the top of the first plot in the respective row. The columns correspond to the different docking algorithms used: 1) Standard rigid-body docking using RosettaDock starting with the antibody crystal structure. 2) Standard rigid-body docking using RosettaDock. 3) Docking with VL-VH optimization. 4) Docking with VL-VH optimization with CDR minimization and CDR H3 perturbation. 5) Docking with SnugDock (VL-VH optimization with CDR minimization and CDR H3+H2 perturbations). 6) Rigid-body docking using EnsembleDock with the ten lowest-energy RosettaAntibody models. 7) Docking using a combined protocol incorporating EnsembleDock and SnugDock with the ten lowest-energy RosettaAntibody models. Refer to Figure 2 legend for explanation of colored points.
(6.97 MB EPS)

Figure S2 Global docking plots. The four letter PDB code at the top of each column indicates the target for which simulations were executed for the respective column. The rows correspond to the different docking algorithms used: 1) Standard rigid body docking using RosettaDock. 2) EnsembleDock 3) SnugDock 4) EnsembleDock-plus-SnugDock. Refer to Figure 2 legend for explanation of colored points.
(1.35 MB TIF)

Table S1 Quantitative accuracy measures of lowest-energy docking decoy for different docking protocols. Refer to Table 1 key for explanation.
(0.03 MB XLS)

Table S2 Quantitative accuracy measures of lowest-energy docking decoy, using WAM homology models. Refer to Table 1 key for explanation.
(0.03 MB XLS)

Table S3 Quantitative accuracy measures of global docking decoys. Refer to Table 1 key for explanation.
(0.03 MB XLS)

Text S1 Rosetta Version Numbers and Command Lines
(0.03 MB DOC)
