# Supplementary figures and images for: SnugDock: Paratope Structural Optimization during Antibody-Antigen Docking Compensates for Errors in Antibody Homology Models
Source: PLoS Comput Biol. 2010 Jan 22;6(1):e1000644. doi: 10.1371/journal.pcbi.1000644 (PMC2800046; doi:10.1371/journal.pcbi.1000644)

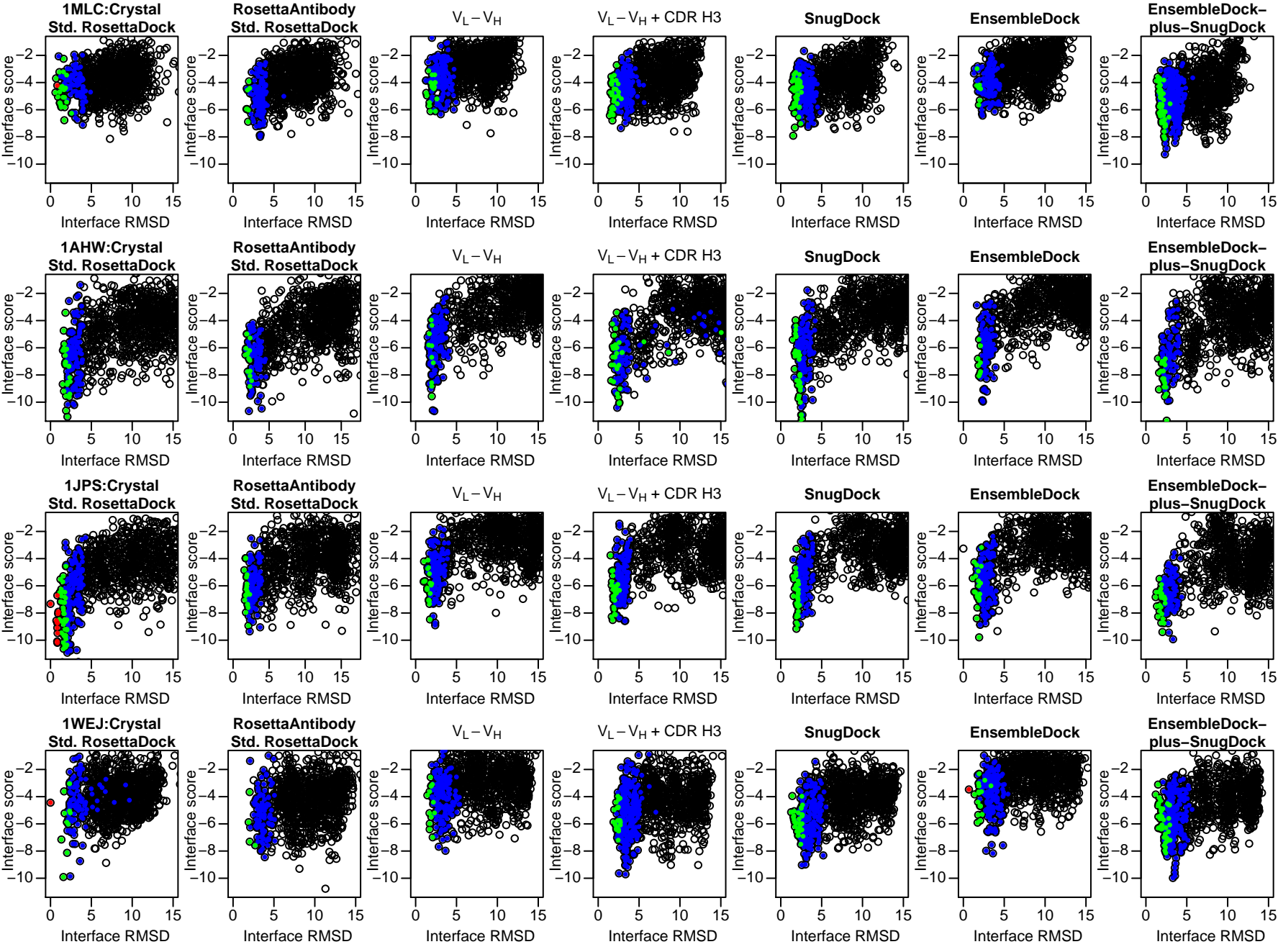

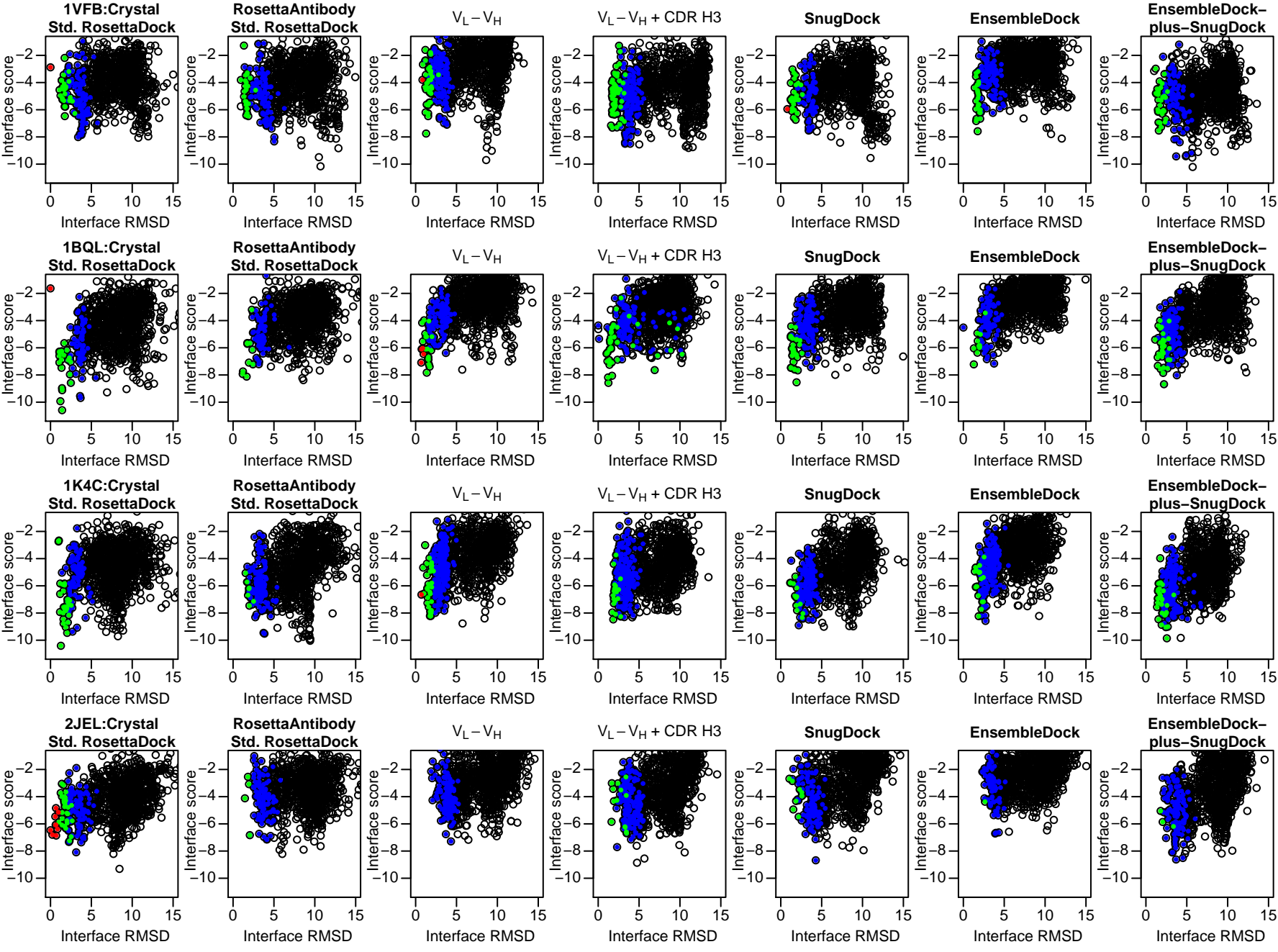

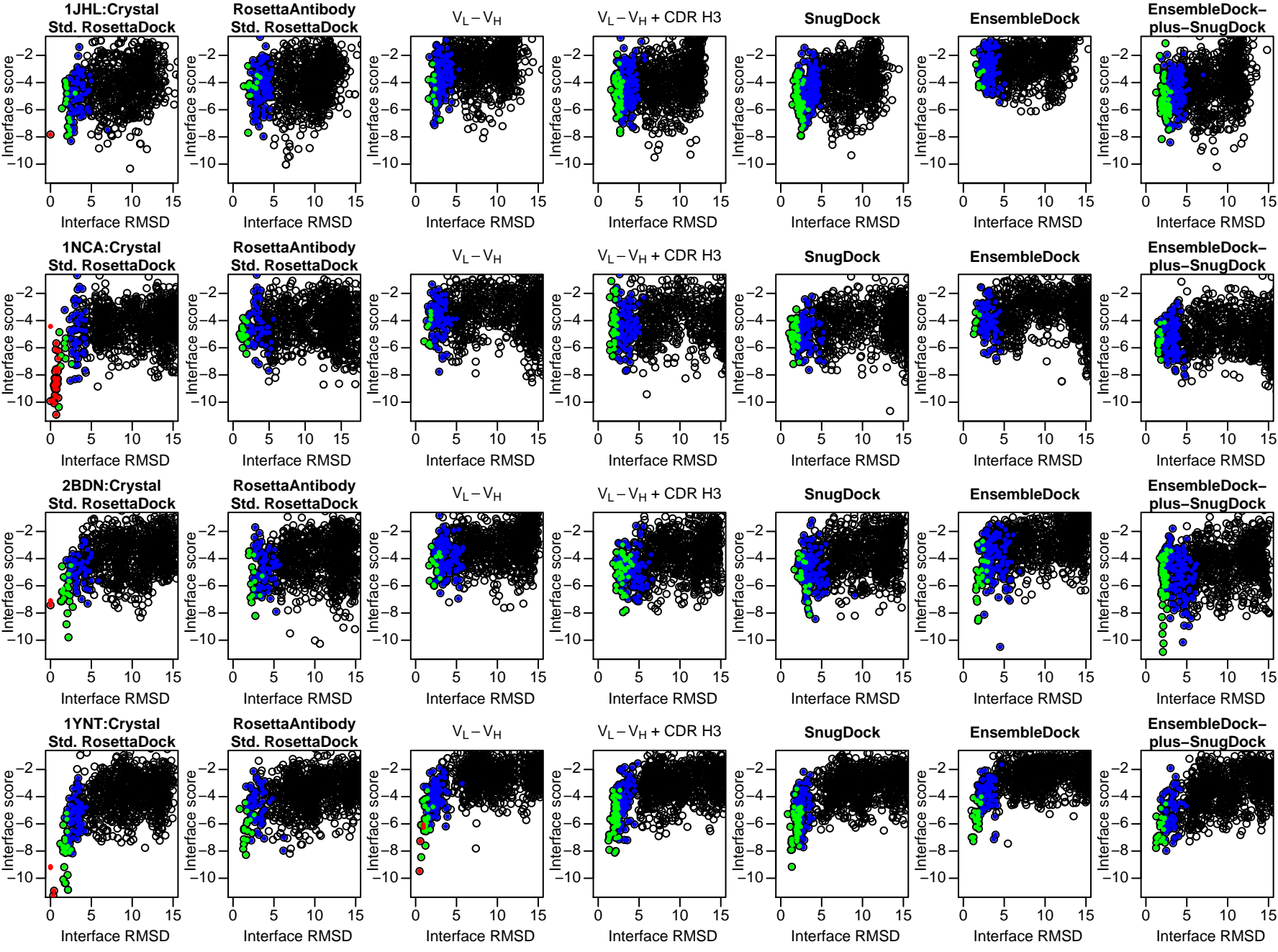

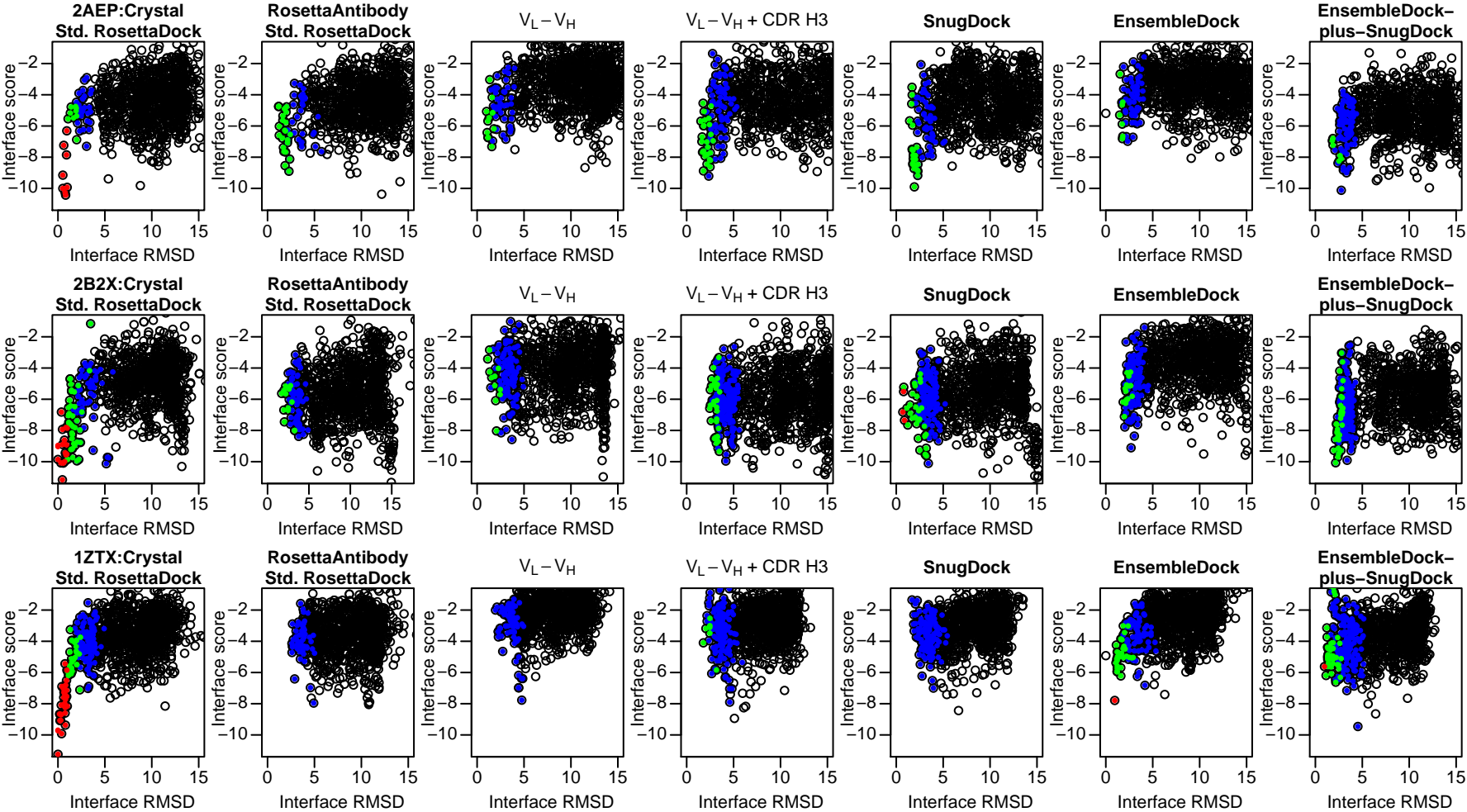

Supplement: Figure S1 — Docking perturbation plots. Each row shows the simulation for one target denoted by the four letter PDB code at the top of the first plot in the respective row. The columns correspond to the different docking algorithms used: 1) Standard rigid-body docking using RosettaDock starting with the antibody crystal structure. 2) Standard rigid-body docking using RosettaDock. 3) Docking with VL-VH optimization. 4) Docking with VL-VH optimization with CDR minimization and CDR H3 perturbation. 5) Docking with SnugDock (VL-VH optimization with CDR minimization and CDR H3+H2 perturbations). 6) Rigid-body docking using EnsembleDock with the ten lowest-energy RosettaAntibody models. 7) Docking using a combined protocol incorporating EnsembleDock and SnugDock with the ten lowest-energy RosettaAntibody models. Refer to Figure 2 legend for explanation of colored points. (6.97 MB EPS) [file pcbi.1000644.s001.pdf]

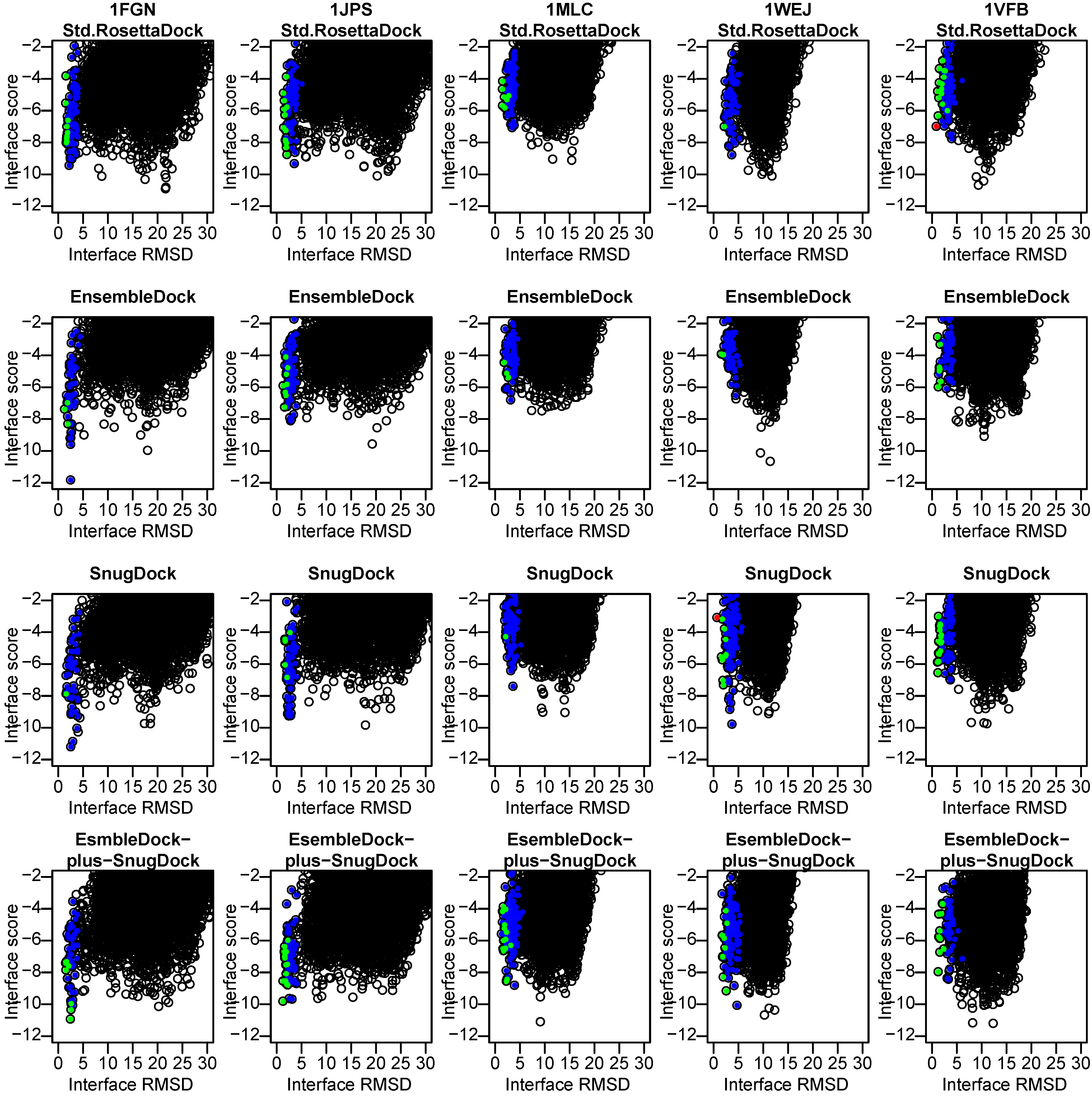

Supplement: Figure S2 — Global docking plots. The four letter PDB code at the top of each column indicates the target for which simulations were executed for the respective column. The rows correspond to the different docking algorithms used: 1) Standard rigid body docking using RosettaDock. 2) EnsembleDock 3) SnugDock 4) EnsembleDock-plus-SnugDock. Refer to Figure 2 legend for explanation of colored points. (1.35 MB TIF) [file pcbi.1000644.s002.tif]
