# Supplementary material for: SnugDock: Paratope Structural Optimization during Antibody-Antigen Docking Compensates for Errors in Antibody Homology Models
Source: PLoS Comput Biol. 2010 Jan 22;6(1):e1000644. doi: 10.1371/journal.pcbi.1000644 (PMC2800046; doi:10.1371/journal.pcbi.1000644)
Supplement: Text S1 — Rosetta Version Numbers and Command Lines (0.03 MB DOC) [file pcbi.1000644.s006.doc]

# Rosetta Version Numbers and Command Lines

## Standard RosettaDock (rigid body docking)

Version Number: 1.2

Command Line:

rosetta.gcc aa 1ABC _ -s 1ABC.ppk -nstruct 1000 -dock -dock_mcm

-dock_pert 3 8 8 -spin -dock_rtmin -ex1 -ex2aro_only -unboundrot

-find_disulf -norepack_disulf -fab1 -use_pdb_numbering

-skip_missing_residues -native 1XYZ -fake_native -quiet

## EnsembleDock

Version Number: 2.3.0

Command Line:

rosetta.gcc aa 1ABC _ -s 1ABC -nstruct 1000 -pose -dock -dock_mcm

-dock_pert 3 8 8 -spin -dock_rtmin -ex1 -ex2aro_only -unboundrot -fab1

-ensemble1 10 -native 1XYZ -fake_native -quiet

## SnugDock

Version Number: 2.3.1

Command Line:

rosetta.gcc aa 1ABC _ -s 1ABC.ppk -nstruct 1000 -pose -dock -dock_mcm

-dock_pert 3 8 8 -spin -dock_rtmin -ex1 -ex2aro_only -unboundrot

-find_disulf -norepack_disulf -fab1 -use_pdb_numbering

-skip_missing_residues -native 1XYZ -snugdock -snugloop -snugh3 -snugh2

-fake_native -quiet

## EnsembleDock-plus-SnugDock

Version Number: 2.3.1

Command Line:

rosetta.gcc aa 1ABC _ -s 1ABC -nstruct 1000 -pose -dock -dock_mcm

-dock_pert 3 8 8 -spin -dock_rtmin -ex1 -ex2aro_only -unboundrot

-find_disulf -norepack_disulf -fab1 -ensemble1 10 -use_pdb_numbering

-skip_missing_residues -native 1XYZ -snugdock -snugloop -snugh3 -snugh2

-fake_native -quiet

**Note:**

1ABC: Denotes the four letter PDB code for the starting structure

1XYZ: Denotes the four letter PDB code for the native (crystal) structure

-unboundrot was used only for unbound antigen
